# The Role of Atypical Ubiquitin Chains in the Regulation of the Antiviral Innate Immune Response

**DOI:** 10.3389/fcell.2019.00392

**Published:** 2020-01-22

**Authors:** Mariska van Huizen, Marjolein Kikkert

**Affiliations:** Department of Medical Microbiology, LUMC Center for Infectious Diseases, Leiden University Medical Center, Leiden, Netherlands

**Keywords:** atypical ubiquitination, K27-linked ubiquitin, innate immune response, antiviral signaling, interferon, NFκB

## Abstract

It is well established that polyubiquitin chains, in particular those linked through K48 and K63, play a key role in the regulation of the antiviral innate immune response. However, the role of the atypical chains linked via any of the other lysine residues (K6, K11, K27, K29, and K33) and the M1-linked linear chains have not been investigated very well yet in this context. This is partially due to a lack of tools to study these linkages in their biological context. Interestingly though, recent findings underscore the importance of the atypical chains in the regulation of the antiviral immune response. This review will highlight the most important advances in the study of the role of atypical ubiquitin chains, particularly in the regulation of intracellular antiviral innate immune signaling pathways. We will also discuss the development of new tools and how these can increase our knowledge of the role of atypical ubiquitin chains.

## Introduction

**FIGURE 1:**
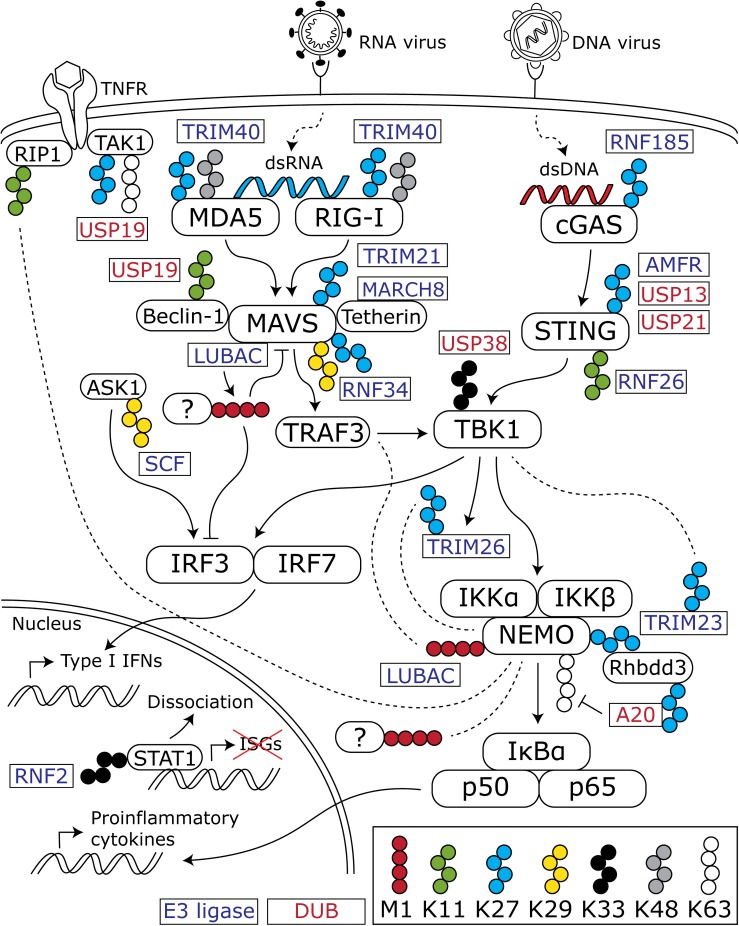
Overview of ubiquitin chains that modulate the antiviral innate immune response. Cytosolic viral nucleic acids are recognized by the dsRNA sensors MDA5 and RIG-I and the dsDNA sensor cGAS. These activate downstream signaling cascades that converge at TBK1 and lead to subsequent activation of the transcription factors IRF3 and -7 and the NFκB subunits p50 and p65. IRF3 and -7 induce the production of type I IFNs, whereas p50 and p65 stimulate proinflammatory cytokine production. In addition to K48- and K63-linked chains, atypical chains are important regulators of the activation and downregulation of the innate immune response. For the sake of clarity, K48- and K63-linked chains are only depicted when an interaction with one of the atypical chains is shown in the discussed literature. Blocks with rounded corners present key innate immune factors, whereas rectangles represent E3 ligases (blue text) and DUBs (red text) that (de)conjugate the indicated chains. Dashed lines indicate an interaction between the connected protein and ubiquitin chains.

Virus infection triggers an immediate response in the host cell, termed the innate immune response. The basic innate immune response pathways, operational in virtually every cell type, have been comprehensively reviewed elsewhere (Schneider et al., 2014; Sparrer and Gack, 2015; Chen et al., 2016). In summary, they comprise a variety of signaling cascades that are initiated by the recognition of pathogen-associated molecular patterns by intra- and extracellular pattern recognition receptors (PRRs). An important class of intracellular PRRs are those that recognize viral nucleic acids in the cytosol. The retinoic acid-inducible gene-I (RIG-I)-like receptors (RLRs) recognize double-stranded RNA (dsRNA), whereas cyclic GMP-AMP synthase (cGAS) recognizes dsDNA. Activation of RLRs and the cGAS-STING pathway leads to a signaling cascade converging at the transcription factors NFκB and IRF3 and -7, which induce the production of proinflammatory cytokines and type I interferons (IFN), respectively (Figure 1).

Ubiquitin plays a crucial role in the activation and downregulation of the innate immune response. Conjugation of ubiquitin onto lysine residues of target proteins by E1, E2, and E3 enzymes and deconjugation by deubiquitinating enzymes (DUBs) can modulate the function, localization, and abundance of the ubiquitinated target (Heaton et al., 2016). Moreover, polyubiquitin chains can be formed by conjugation of a subsequent ubiquitin molecule to one of the lysine residues or the N-terminal methionine of the previous ubiquitin molecule. These polyubiquitin chains have different topologies, thereby creating a complex ubiquitin code that can direct many different outcomes (Komander and Rape, 2012; Kulathu and Komander, 2012). Regulation of the innate immune response by polyubiquitination is well characterized for K48- and K63-linked chains (reviewed in Davis and Gack, 2015). However, knowledge of the role of the atypical linkages, linked via any of the other lysine residues or the N-terminal methionine, is still rather limited. Here we focus on the role of linear, K11-, K27-, K29-, and K33-linked chains in the innate immune response and the tools that are available to study these chains. Table 1 summarizes the functions of atypical ubiquitination in innate immune responses, and associated E3 enzymes and DUBs, as will be discussed below.

**TABLE 1:** Overview of the functions of atypical ubiquitination and the associated E3 enzymes and DUBs.

**Ubiquitin linkage**	**Modifying enzyme**	**Substrate**	**Functional outcome**	**References**
Linear	LUBAC^a^	?	Interaction of NEMO with linear chains potentiates NFκB activation.	Rahighi et al., 2009; Hadian et al., 2011; Kensche et al., 2012
		NEMO	Upregulates NFκB activation and disrupts MAVS-TRAF3 interaction, thereby inhibiting IRF3 activation and the IFN response.	Tokunaga et al., 2009; Belgnaoui et al., 2012
		?	Interaction of MAVS with LUBAC leads to the formation of linear chains that disrupt the MAVS signalosome and prevent downstream signaling.	Khan et al., 2016
K11	RNF26^a^	STING	Inhibits STING degradation, leading to increased type I IFN and cytokine production.	Qin et al., 2014
	USP19^b^	Beclin-1	Stabilizes Beclin-1 and limits type I IFN production by disrupting the interaction between RIG-I and MAVS.	Jin et al., 2016
	?	RIP1	Interacts with NEMO.	Dynek et al., 2010
K27	TRIM23^a^	NEMO	Leads to NFκB and IRF3 activation.	Arimoto et al., 2010
		NEMO and Rhbdd3	Recruits the DUB A20 to remove K63-linked chains from NEMO, thereby preventing excessive NFκB activation.	Liu et al., 2014
	TRIM23^a^	TRIM23	Activates TBK1 and thereby induces antiviral autophagy.	Sparrer et al., 2017
	TRIM26^a^	TRIM26	Interacts with NEMO, leading to increased type I IFN and cytokine production.	Ran et al., 2016
	TRIM40^a^	RIG-I and MDA5	Induces proteasome-mediated degradation of RIG-I and MDA5, thereby inhibiting the type I IFN response.	Zhao et al., 2017
	TRIM21^a^	MAVS	Enhances type I IFN production.	Liu H. et al., 2018; Xue et al., 2018
	MARCH8^a^	MAVS	Induces autophagy-mediated degradation of MAVS, thereby restricting the type I IFN response.	Jin et al., 2017
	RNF185^a^	cGAS	Induces IRF3 activation and the production of type I IFNs and proinflammatory cytokines.	Wang et al., 2017
	AMFR^a^	STING	Recruits TBK1 to STING and induces IRF3 activation and the production of type I IFNs and proinflammatory cytokines.	Wang Q. et al., 2014
	USP13 and USP21^b^	STING	Inhibits IRF3 activation and the production of type I IFNs and proinflammatory cytokines.	Chen et al., 2017; Sun et al., 2017
	USP19^b^	TAK1	Inhibition of proinflammatory cytokine production.	Lei et al., 2019
K27 and K29	RNF34^a^	MAVS	Induces autophagy-mediated degradation of MAVS, thereby restricting the type I IFN response.	He et al., 2019
K29	SKP1-Cullin-Fbx21^a^	ASK1	Induces IFNβ and IL-6 production.	Yu et al., 2016
K33	USP38^b^	TBK1	Prevents TBK1 degradation and induces IRF3 activation.	Lin et al., 2016
	RNF2^a^	STAT1	Suppresses ISG transcription.	Liu S. et al., 2018

*This table summarizes the E3 enzymes and DUBs that (de)conjugate atypical ubiquitin chains in the context of the innate immune response and the effects of (de)conjugation of these chains on the innate immune response. ^*a*^Indicates that the modifying enzyme is an E3 ligase, whereas ^*b*^indicates that the enzyme is a DUB.*

## Linear Chains Are Important Regulators of NEMO and NFκB Signaling

Since the discovery of the linear ubiquitin chain assembly complex (LUBAC) that uniquely catalyzes the formation of linear chains, it has become evident that LUBAC and linear chains are crucial for the activation of nuclear factor κB (NFκB) signaling (Kirisako et al., 2006; Gerlach et al., 2011; Tokunaga et al., 2011). Linear chains are especially important for tumor necrosis factor α (TNFα) signaling, but are also involved in other immune signaling pathways (Spit et al., 2019). One important mechanism in the activation of NFκB, is the interaction of linear chains with NFκB essential modulator (NEMO). NEMO is part of the IKK complex that phosphorylates NFκB inhibitor α (IκBα), thereby releasing the NFκB subunits p50 and p65, which then act as transcription factors and induce the transcription of proinflammatory cytokines. The UBAN domain (ubiquitin binding in ABIN proteins and NEMO) of NEMO has a strong binding-preference for linear chains, although some studies indicate that it can also bind longer K63-linked chains. NEMO mutants that cannot bind linear chains or NEMO chimeras in which the UBAN domain is replaced by the NZF domain of TAB2, a ubiquitin-binding domain (UBD) that binds specifically to K63-linked chains, cannot activate NFκB upon TNFα stimulation (Rahighi et al., 2009; Hadian et al., 2011; Kensche et al., 2012). Altogether, these studies show that NEMO UBAN has a strong preference for linear chains, and that this is required and sufficient for NFκB activation.

In addition to its binding to linear chains, NEMO is also a substrate for the conjugation of linear chains by LUBAC (Tokunaga et al., 2009). Furthermore, association of LUBAC with NEMO mediates the interaction between NEMO and TRAF3, which then leads to the disruption of the MAVS-TRAF3 complex. This results in NFκB activation and inhibition of type I IFN signaling (Belgnaoui et al., 2012). LUBAC has also been found to interact with MAVS. Hepatitis B virus-induced recruitment of the E3 ligases Parkin and LUBAC to MAVS leads to the formation of linear chains. Interaction of MAVS with these chains results in a disruption of the MAVS signalosome and downstream IRF3 activation, thereby inhibiting the type I IFN response. It is unclear to which substrate these chains are conjugated (Khan et al., 2016).

In summary, linear chains potentiate NFκB signaling, while inhibiting type I IFN signaling.

## K11-Linked Chains Regulate the Degradation of Innate Immune Factors

K11-linked ubiquitination is associated with the regulation of the cell cycle and proteasome-mediated degradation (Meyer and Rape, 2014; Grice and Nathan, 2016; Yau et al., 2017). By regulating the degradation of innate immune factors, K11-linked ubiquitination can affect the innate immune response. For example, RNF26-mediated K11-linked ubiquitination of STING causes inhibition of STING degradation. Thereby, the production of type I IFNs and proinflammatory cytokines is potentiated (Qin et al., 2014). On the other hand, RNF26 can induce autophagy-mediated degradation of IRF3, which limits the production of type I IFNs. This is dependent on the E3 ligase activity of RNF26, but the authors could not identify which ubiquitin linkage is involved (Qin et al., 2014). Overall, it seems that RNF26, partially via K11-linked ubiquitination, can both prevent and promote the induction of type I IFNs via the degradation of its target, and that this is under strict temporal regulation.

The presence of K11- and K48-linked chains on Beclin-1, a protein interacting with MAVS, has been associated with proteasome-mediated degradation of Beclin-1 (Jin et al., 2016). Removal of K11-linked chains by the DUB USP19 prevents this and leads to Beclin-1 stabilization. Stabilized Beclin-1 induces autophagy and inhibits the interaction between RIG-I and MAVS, thereby limiting the production of type I IFNs upon SeV or vesicular stomatitis virus (VSV) infection. This way, K11-linked ubiquitination of Beclin-1 indirectly inhibits autophagy and promotes the type I IFN response by inducing Beclin-1 degradation (Jin et al., 2016).

Lastly, there is some evidence that NEMO can bind K11-linked chains, which are for example conjugated to receptor-interacting serine/threonine-protein kinase 1 (RIP1), a kinase associated with the TNFα receptor (Dynek et al., 2010). However, it is unclear what the effects of this interaction are.

## K27-Linked Chains: Balancing Activation and Inhibition?

It is becoming more and more evident that K27-linked chains are important regulators of the innate immune response. The first evidence for this came from a study by Arimoto et al. (2010). They showed that E3 ligase TRIM23 can conjugate K27-linked chains to NEMO and that this is required for the induction of NFκB and IRF3 upon activation of RLR signaling (Arimoto et al., 2010). K27-linked chains on NEMO subsequently serve as an interaction platform for other factors that regulate the innate immune response. This is for example illustrated by binding of Rhbdd3, a serine protease that regulates epidermal growth factor signaling, to K27-linked chains on NEMO. This leads to K27-linked ubiquitination of Rhbdd3 and recruitment of the DUB A20. A20 then removes K63-linked chains from NEMO, thereby preventing excessive NFκB activation. By this mechanism, Rhbdd3 was shown to control the activation of dendritic cells and to limit Th17 cell-mediated colitis in mice (Liu et al., 2014).

TRIM23 is also auto-ubiquitinated with K27-linked chains. As a result, TRIM23 activates TBK1 by its GTPase activity. TBK1 subsequently phosphorylates the selective autophagy receptor p62, which leads to the induction of autophagy upon infection with several different DNA and RNA viruses (Sparrer et al., 2017).

Another E3 ligase that is auto-ubiquitinated with K27-linked chains is TRIM26. Upon activation of RLR signaling, TBK1 phosphorylates TRIM26, leading to TRIM26 auto-ubiquitination. NEMO then interacts with the K27-linked chains conjugated to TRIM26, which induces the expression of proinflammatory cytokines, type I IFNs, and interferon stimulated genes (ISGs) (Ran et al., 2016).

Another E3 ligase of the TRIM family, TRIM40, was shown to conjugate K27- and K48-linked chains to the dsRNA sensors RIG-I and Melanoma Differentiation-Associated protein 5 (MDA5). This leads to attenuation of RNA virus-induced RLR signaling. Mechanistically, TRIM40-mediated ubiquitination of RIG-I and MDA5 induces proteasome-mediated degradation of these proteins (Zhao et al., 2017). Therefore, the authors conclude that both K27- and K48-linked chains are involved in proteasome-mediated degradation. However, they do not discriminate between the functions of these two linkages. Since K48-linked chains have strongly been linked to proteasome-mediated degradation, it may be likely that the proteasome-mediated degradation could be attributed to K48-linked ubiquitination, while the role of K27-linked chains in degradation of RIG-I and MDA5 remains unclear.

Lastly, TRIM21 has been suggested to catalyze K27-linked ubiquitination of MAVS (Liu H. et al., 2018; Xue et al., 2018). TRIM21 expression is induced by infection with different RNA viruses and it potentiates the innate immune response (Liu H. et al., 2018; Xue et al., 2018). These studies clearly demonstrate that TRIM21 has antiviral effects. However, the presented Western blots which show that TRIM21 exerts its effects via K27-linked ubiquitination are not very convincing, and this should be further investigated.

Another E3 ligase that can conjugate K27-linked chains to MAVS, is MARCH8 (Jin et al., 2017). MARCH8 is recruited to MAVS by Tetherin, an ISG that restricts the release of enveloped viruses (Evans et al., 2010). Recruitment of MARCH8 by Tetherin induces K27-linked ubiquitination of MAVS followed by the degradation of MAVS by selective autophagy. This provides a negative feedback loop by which the innate immune response is restricted (Jin et al., 2017). Another E3 ligase that induces autophagic degradation of MAVS, is RNF34. RNF34 catalyzes both K27- and K29-linked ubiquitination of MAVS (He et al., 2019). However, the authors also show that RNF34 is important for the clearance of damaged mitochondria by mitophagy, so the question is whether the degradation of MAVS is specific or is a result of mitophagy (He et al., 2019).

RNF185-mediated K27-linked ubiquitination of cGAS, and AMFR-mediated K27-linked ubiquitination of STING both lead to the induction of a proinflammatory and antiviral response upon stimulation with different DNA ligands or infection with the DNA virus herpes simplex virus 1 (HSV-1). K27-linked ubiquitination of cGAS and STING is required for TBK1 activation (Wang Q. et al., 2014; Wang et al., 2017). Mechanistically, K27-linked chains on STING are responsible for the recruitment of TBK1 to STING (Wang Q. et al., 2014). The DUBs USP13 and USP21 were shown to remove K27-linked ubiquitin from STING (Chen et al., 2017; Sun et al., 2017). These studies confirmed that K27-linked ubiquitin activates the immune response upon infection with several DNA viruses or the intracellular bacterium *Listeria monocytogenes* (Chen et al., 2017; Sun et al., 2017).

TGFβ-activated kinase 1 (TAK1) is a protein that is activated by various inflammatory stimuli and subsequently induces activation of NFκB signaling. TAK1 activation is strongly regulated by posttranslational modifications, including K48- and K63-linked ubiquitination (Hirata et al., 2017). Recently, it was shown that TAK1 can also be K27-linked ubiquitinated and that both K27- and K63-linked chains can mediate the interaction with TAK1-binding protein 2 (TAB2) and TAB3. Removal of K27- and K63-linked chains by USP19 inhibited TNFα- and IL-1β-induced NFκB activation, suggesting that these ubiquitin chains normally activate TAK1 downstream signaling (Lei et al., 2019). However, the authors could not discriminate between the role of K27- and K63-linked chains, due to technical constraints.

In summary, K27-linked chains are important activators of the innate immune response, in this context often conjugated by members of the TRIM family but also by other E3 ligases. These chains are also part of negative feedback loops that prevent excessive inflammation and immunopathology, hence K27-linked ubiquitin chains could be used to give a temporary controlled boost to the innate immune system, when this is deemed necessary by the cell.

## K29-Linked Chains on ASK1 Activate Irf3

Very little is known about the role of K29-linked ubiquitination in the innate immune response. It has been shown that the SKP1-Cullin-Fbx21 (SCF) E3 ligase complex is activated upon VSV and HSV-1 infection. This complex then catalyzes K29-linked ubiquitination of apoptosis signal-regulating kinase 1 (ASK1), thereby inducing phosphorylation of JNK1/2, p38, and IRF3, and activation of the transcription factor activator protein-1 (AP-1). Altogether, this leads to the production of IFNβ and interleukin-6 (Yu et al., 2016). However, it remains to be elucidated how virus infection leads to the activation of ASK1 signaling.

## K33-Linked Chains Modulate RLR and Type I IFN Signaling

K33-linked ubiquitination is associated with cGAS-STING- and RLR-induced type I IFN signaling. Upon infection with different DNA and RNA viruses, TBK1 is K33-linked ubiquitinated, which leads to IRF3 activation (Lin et al., 2016). This can be reversed by the DUB USP38. USP38-mediated removal of K33-linked ubiquitin is associated with an increase in K48-linked ubiquitination and subsequent proteasome-mediated degradation of TBK1, thereby downregulating the antiviral response (Lin et al., 2016). Another study describes K33-linked ubiquitination of the type I IFN-induced transcription factor STAT1. This is mediated by the E3 ligase RNF2. Upon interferon stimulation, RNF2 binds to STAT1 in the nucleus and mediates K33-linked ubiquitination of the STAT1 DNA binding domain. This leads to the dissociation of STAT1 from the promotor of several ISGs, thereby suppressing the production of ISGs (Liu S. et al., 2018). These two studies demonstrate two different ways in which K33-linked chains can be involved in the regulation of the innate immune response. Further studies are necessary to elucidate how these mechanisms complement each other and regulate RLR and interferon signaling.

## Tools to Study Specific Ubiquitin Linkages in Their Biological Context

Probably the most reliable technique to identify specifically linked ubiquitin chains on a purified substrate or in the total cellular ubiquitin pool, is using mass spectrometry. However, this is relatively elaborate, and may not be available to all researchers. Furthermore, this does not allow the identification of specific ubiquitin linkages conjugated to a specific substrate in cells. Most biochemical studies that try to identify specific ubiquitin linkages therefore rely on expression of ubiquitin mutants that contain only one lysine residue (KX-only mutants) or individual lysine-to-arginine substitutions (KXR mutants). These are then individually co-transfected into cells together with the other proteins of interest. However, using this approach it is hard to study the role of a specific ubiquitin linkage in the innate immune response, as most cultured cells, such as the often-used 293T cells, have important deficiencies in these pathways (Burdette et al., 2011; Lin et al., 2014). Therefore, one should use cells that have an intact innate immune system, however, transfection of these cells is usually rather inefficient and subsequent virus infection is very hard. Another frequently used method are *in vitro* ubiquitination and deubiquitination assays. Although these can be a helpful tool, such assays do not take into account the subcellular localization of the proteins involved and do not allow to study the effects of a specific chain on a specific target in the innate immune response. Therefore, methods are needed to directly detect specific ubiquitin linkages in cells. For linear, K11-, K27-, K48-, and K63-linked chains, linkage-specific antibodies have been generated (Newton et al., 2008; Matsumoto et al., 2010, 2012). These have been used with varying results, and in most cases they hardly produce any specific signal when used in cell lysates. The generation of linkage-specific antibodies is apparently very challenging, which is probably due to the sometimes very subtle structural differences between different ubiquitin chains.

An alternative to antibodies are affimers. These are small scaffold proteins of which the sequence is based on a phytocystatin consensus sequence (Tiede et al., 2014, 2017). The insertion of two variable peptide regions into this sequence was used to construct a phage-display library that can be screened for any protein of interest (Tiede et al., 2014). Michel et al. (2017) have described the development of an affimer against K6-linked ubiquitin. This affimer was used successfully in pull downs, Western blotting, and confocal microscopy (Michel et al., 2017). Using the affimer, the cellular E3 ligase that catalyzes K6-linked ubiquitination, a DUB with strong preference for K6-linked ubiquitin and a substrate could be identified (Gersch et al., 2017; Michel et al., 2017; Heidelberger et al., 2018). In addition, an affimer against K33-linked ubiquitin was developed. However, this affimer also recognized K11-linked ubiquitin (Michel et al., 2017). Most likely this is the result of heterogeneity in the conformation of polyubiquitin chains, which is why chains linked via different residues can have closely resembling conformations (Wang Y. et al., 2014). Although this shows that it can be hard to achieve linkage-specificity, affimers could be a powerful alternative for antibodies.

In addition to methods that directly detect a specific type of ubiquitin chain, linkage-specific DUBs can be used to discriminate between different linkages in a cell lysate or on a target that was precipitated using pull-downs. The following linkage-specific DUBs are available: OTULIN for linear chains, Cezanne for K11-linked chains, Otubain-1 for K48-linked chains, and AMSH or OTUD1 for K63-linked chains (Mevissen et al., 2013). No DUBs are known that have specificity for K6-, K27-, K29-, and K33-linked chains. However, OTUD3 and USP30 have a strong preference for K6- and K11-linked chains, whereas TRABID has a strong preference for K29- and K33-linked chains. When OTUD3 or USP30 are used in combination with Cezanne, the discrimination between K6- and K11-linked chains can be made (Mevissen et al., 2013; Cunningham et al., 2015). Based on this principle, a method was developed termed ubiquitin chain restriction (UbiCRest) in which *in vitro* ubiquitinated proteins, cell lysates, or precipitated immunocomplexes are incubated with a combination of the aforementioned linkage-specific DUBs (Hospenthal et al., 2015).

The UBDs of linkage-specific DUBs and other proteins that interact with specific ubiquitin linkages can also be exploited as biosensors. TRABID has 3 NZF domains that can bind a variety of different ubiquitin chains. The NZF1 domain specifically binds K29- and K33-linked chains (Kristariyanto et al., 2015). This NZF1 domain was used to pull down polyubiquitin chains from cells. Subsequently, the immunocomplexes were treated with the Crimean-Congo Hemorrhagic Fever virus OTU (vOTU) DUB to discriminate between K29- and K33-linked chains (Akutsu et al., 2011; Kristariyanto et al., 2015). According to the authors, vOTU cleaves all types of ubiquitin chains except for K29-linked chains (Kristariyanto et al., 2015). Contrary to this, there is also evidence that vOTU cleaves all linkages except for linear chains (Mevissen et al., 2013). Using their approach, the authors showed that K29-linked ubiquitin can be part of heterotypic chains containing also K48-linked ubiquitin (Kristariyanto et al., 2015). Two other biosensors have been described, one that is based on the UBAN domain of NEMO and recognizes linear chains and one that is based on the NZF domain of TAB2 and recognizes K63-linked chains. These domains were coupled to GFP and could thereby be used in microscopy and live cell imaging (van Wijk et al., 2012; Greenfeld et al., 2015). Although these biosensors are a valuable tool, their development depends on the availability of a UBD that specifically binds to a certain ubiquitin linkage.

Another method to obtain insight in the cellular function of a specific ubiquitin linkage has been developed by Xu et al. (2009). They developed a tetracycline-inducible RNAi system with which the expression of all four ubiquitin genes can be knocked down and replaced by a KXR mutant. Using cells expressing K63R ubiquitin, they could show that K63-linked chains are required for IKK activation, but only by IL-1β and not by TNFα (Xu et al., 2009). Although this setup is laborious to create and leads to a general depletion of a specific ubiquitin linkage, this strategy can be very useful in elucidating the role of a certain linkage in the innate immune response or any other signaling cascade of interest.

In summary, for M1-, K48- and K63-linked chains rather well-functioning antibodies, linkage-specific DUBs, and UBD-based biosensors exist, whereas for most of the other linkages, including K27- and K33-linked chains, very few or no tools are available. Potentially, new UBDs could be developed based on the structure of UBDs in complex with ubiquitin chains for which no specific UBD is known. By structure-guided mutagenesis, it would in theory be possible to develop new biosensors that recognize for example K27- or K33-linked chains.

## Conclusion

The innate immune response is a crucial first line of defense against virus infection and is responsible for the recruitment of innate immune cells to the site of infection and the induction of the adaptive response. However, overactivation of the innate response can lead to excessive inflammation and immunopathology. Therefore, activation of the innate immune response is subject to strong regulation. Besides phosphorylation, this is strongly mediated by ubiquitination. The variety in ubiquitin chains, each with their unique properties, enables very precise fine-tuning of the innate immune response. Some linkages, such as linear chains, are currently almost exclusively linked to the innate immune response. However, most linkages are involved in many different processes. K27-linked chains seem to function mainly as activators of the innate immune response, although they can also have inhibitory effects. For K29- and K33-linked ubiquitin, too little data is available to define whether they have a specific role in the innate immune response. In addition to these homotypic chains that are linked via one specific lysine residue, hybrid or mixed chains exist as well (Akutsu et al., 2016). M1/K63-linked hybrid chains can serve as unique scavengers that recruit TAK1, IKKα, and IKKβ via the K63 linkage, and NEMO via the M1 linkage (Emmerich et al., 2013). Overall, the ubiquitin code has a fascinating complexity and elucidating more of this will give us important insight into the intricate interactions that regulate the innate immune response.

## Author Contributions

MH wrote the original draft of the manuscript. MH and MK contributed to the manuscript revision, and read and approved the submitted version.

## Conflict of Interest

The authors declare that the research was conducted in the absence of any commercial or financial relationships that could be construed as a potential conflict of interest.
